# Tackling Structures of Long Noncoding RNAs

**DOI:** 10.3390/ijms141223672

**Published:** 2013-12-04

**Authors:** Irina V. Novikova, Scott P. Hennelly, Karissa Y. Sanbonmatsu

**Affiliations:** Los Alamos National Laboratory, Los Alamos, NM 87545, USA; E-Mails: ivn@lanl.gov (I.V.N.); sph@lanl.gov (S.P.H.)

**Keywords:** long noncoding RNAs, lncRNAs, secondary structure, chemical probing, SHAPE, epigenetics

## Abstract

RNAs are important catalytic machines and regulators at every level of gene expression. A new class of RNAs has emerged called long non-coding RNAs, providing new insights into evolution, development and disease. Long non-coding RNAs (lncRNAs) predominantly found in higher eukaryotes, have been implicated in the regulation of transcription factors, chromatin-remodeling, hormone receptors and many other processes. The structural versatility of RNA allows it to perform various functions, ranging from precise protein recognition to catalysis and metabolite sensing. While major housekeeping RNA molecules have long been the focus of structural studies, lncRNAs remain the least characterized class, both structurally and functionally. Here, we review common methodologies used to tackle RNA structure, emphasizing their potential application to lncRNAs. When considering the complexity of lncRNAs and lack of knowledge of their structure, chemical probing appears to be an indispensable tool, with few restrictions in terms of size, quantity and heterogeneity of the RNA molecule. Probing is not constrained to *in vitro* analysis and can be adapted to high-throughput sequencing platforms. Significant efforts have been applied to develop new *in vivo* chemical probing reagents, new library construction protocols for sequencing platforms and improved RNA prediction software based on the experimental evidence.

## 1. Introduction

Our understanding of RNA roles in the cell has expanded significantly over the past decade. Currently, RNA molecules are viewed as important catalytic molecular machines and important regulators of gene expression during transcription, mRNA maturation and translation [1–3]. In recent years, long noncoding RNAs (lncRNAs) have emerged as key players in higher eukaryotes, producing new insights into evolution, development and disease [4–8]. LncRNAs have been implicated in the regulation of transcription factors and chromatin-remodeling complexes [1,9]. Several lncRNAs also interact directly with promoter regions [10]. LncRNAs also act as miRNA scavengers, regulators of hormone receptors and modulators of alternative splicing events [11–14].

Certain functional properties of RNA are determined by primary structure (*i.e.*, sequence specific processes). For example, mature miRNA and siRNA molecules basepair to their targets [15]. In addition to primary structure, RNA has a unique ability to adopt a variety of complex secondary and tertiary folds. Many sequence-specific and structural features have been characterized to date [16–18]. The structural versatility of RNA allows it to perform a variety of functions, ranging from precise protein recognition to catalysis and metabolite sensing [19,20].

Ribosomal RNAs, transfer RNAs, and riboswitches have long been the focus of structural studies, producing numerous experimental and theoretical methods in addition to the structures themselves [21,22]. Among the various classes of RNAs, lncRNAs remain the least characterized in terms of function and structure. In this review, we cover the current methodologies used to tackle RNA structure, discuss major innovations in these methodologies and comment on future directions, focusing on lncRNAs.

## 2. RNA Structure

RNA folding often proceeds in a hierarchical fashion, from initial secondary structure formation to tertiary collapse [23]. Although some exceptions may exist [24], many RNAs are thought to undergo this general order of events during folding. We note that RNA chaperones and other RNA-binding factors may significantly influence the RNA folding pathway [25].

**Figure 1.**
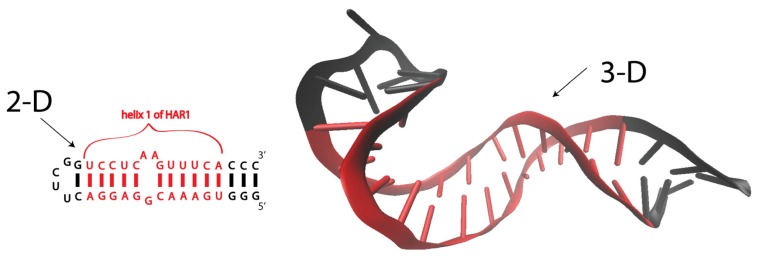
Secondary (2-D) and tertiary (3-D) folds of helix 1 of human HAR1 RNA. Nucleotides, denoted in red, belonging to a native helical region of HAR1 RNA. Nucleotides, denoted in black, are added in order to stabilize the entire construct for NMR. The NMR structure is adapted from Ziegeler *et al*. [26].

The secondary structure of RNA is established by the combination of complementary GC and AU Watson-Crick basepairs, as well as GU wobble pairs (Figure 1). RNA secondary structures are often summarized in a 2-D diagram of RNA helices, connected by single-stranded regions, including terminal loops, internal loops, bulges and junctions. Helices and single-stranded regions may also facilitate the formation of long-range interactions. Long-range tertiary interactions include non-canonical basepairs, base triples, loop-loop interactions, pseudoknot interactions and unique tertiary motifs (e.g., receptor-loops). The abovementioned tertiary contacts combined with cation interactions (Mg^2+^, K^+^) specify the precise geometrical and topological arrangements of the helices, yielding the three-dimensional architecture, or tertiary fold (Figure 1).

Determining the RNA secondary fold is the first step towards understanding the basic principles of RNA function. 2-D RNA maps often guide researchers in the design of successful mutation, deletion and insertion experiments for *in vivo* studies of RNA function. In combination with protein footprinting studies, 2-D information can also aid in understanding RNA-protein motifs and RNA structural requirements for the RNA-protein interactions. Alternations in 2-D structure can provide the essential clues necessary to unlock mechanism. For instance, the mechanism of SAM-I riboswitch has been attained by the secondary structure studies only, and later validated by SAXS and X-ray [27–29].

## 3. Approaches to Determine 2-D Structures

**Figure 2.**
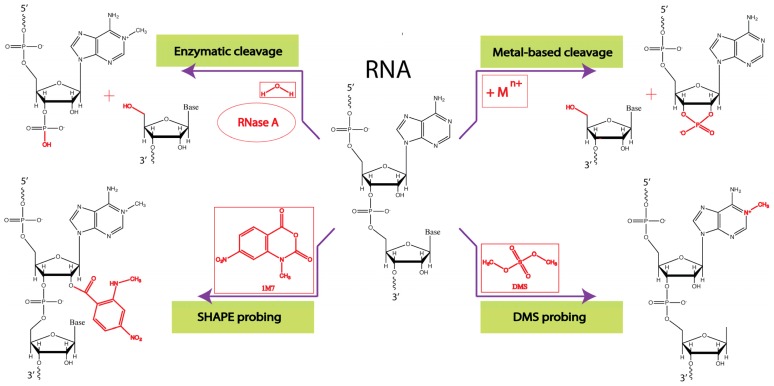
Mechanistic examples of enzymatic and chemical probing of RNA structure. These include: (i) RNase A cleavage of the RNA backbone; (ii) metal-assisted cleavage of the RNA backbone with the subsequent formation of a 2′,3′-cyclic phosphate product; (iii) the methylation of adenine by dimethylsulfate (DMS); and (iv) a 2′-adduct formation with the SHAPE reagent (1M7). Chemical groups important for each reaction are labeled.

**Figure 3.**
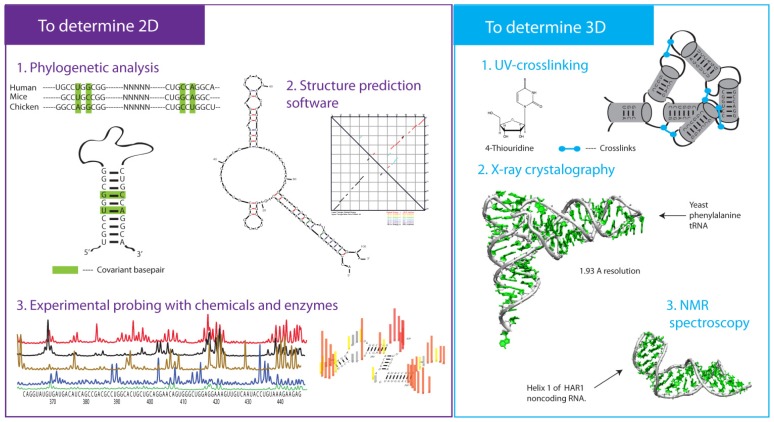
Summary of common strategies to tackle RNA structure. Left (purple), common approaches to determine the secondary fold of RNA. Right (blue), common methods to determine RNA tertiary fold. Adaptations of the crystal structure of tRNA and NMR structure of helix H1 of HAR1 are shown [26,44].

There are three major experimental approaches to dissect the RNA secondary profile: enzymatic probing, chemical probing and covariance analysis of sequence alignments across diverse organisms (Figures 2 and 3).

### 3.1. Enzymatic Probes

The first experimental methods to tackle RNA structure utilized nucleases. The majority of nucleases cleave specifically or more rapidly the single-stranded regions of RNA (*i.e.*, regions not constrained by basepairing). Nuclease S1, nuclease P1, RNase T1, RNase U2, and RNase A are enzymatic probes commonly used to map single stranded RNA (Figure 2). RNase V1 is the enzyme of choice used to study double stranded RNA regions [30,31]. Due to the high-molecular weight of these enzymes, the efficiency of cleavage depends on RNA solvent accessibility [32]. RNase V1 probing is a popular method, directly interrogating helical regions. RNase V1 is also known to cleave stacked, but single-stranded nucleotides [31].

### 3.2. Chemical Probes

There is a wide spectrum of chemical probes available today [33]. Metal ions are the simplest chemical probes, which promote RNA cleavage. In particular, the cleavage of unconstrained nucleotides, which can sample the optimal “in-line” geometry, is facilitated by this mechanism (Figure 2) [34]. Mg^2+^-assisted in-line probing has been widely used to study riboswitches [34,35]. Many probes used today act by modifying RNA molecules rather than assisting in RNA cleavage. While modified RNA nucleotides cannot serve as templates for reverse transcriptases, the modification sites themselves are recognized as stops in the primer extension reaction. The degree of modification is directly correlated with the nucleotide reactivity towards this reagent.

Until recently, other widely-used chemical probes included: dimethyl sulfate (DMS), kethoxal and 1-cyclohexyl-(2-morpholinoethyl)carbodiimide metho-*p*-toluenesulfonate (CMCT) (Figure 2). These reagents are base-specific: DMS reacts with single-stranded adenine and cytosine, kethoxal modifies guanosine, and CMCT primarily targets uracil. More recently, the SHAPE reagents developed by K. Weeks and co-workers have proven extremely useful [36–38]. While the DMS, kethoxal and CMCT reagents target the base of the nucleotide, SHAPE probes react with the RNA backbone, probing its mobility (Figure 2). This is an important advantage, allowing all four nucleotides to be probed in a single experiment. The current spectrum of SHAPE reagents includes NMIA (*N*-methylisatoic anhydride), 1M7 (1-methyl-7-nitroisatoic anhydride) and BzCN (benzoyl cyanide), which vary in hydrolysis half-lives. More detailed information on advances in chemical probing can be found elsewhere [39].

### 3.3. Phylogenetic Analysis

The most powerful and reliable alternative method to determine the helical composition of RNA is comparative sequence analysis (Figure 3). This method was initially demonstrated on the 5S ribosomal RNA [40]. Subsequently, in combination with enzymatic probing, this approach was used to produce the first secondary profiles of 16S and 23S ribosomal RNAs [41–43]. This phylogenetic approach relies on the alignment of a large number and diverse homologous sequences. The basepairs that interconvert between GC and AU in various organisms are called covariant.

This technique has proven quite powerful in the case of ribosomal and riboswitch RNAs, where several thousand sequences are available and covariance analysis can be used to predict the presence of helices with high confidence. LncRNAs, however, are present primarily in the mammalian transcriptome, limiting the number of available sequences for use in multiple sequence alignments. In addition, many lncRNAs exhibit very low sequence conservation, making multiple sequence alignment difficult. Finally, lncRNAs often exhibit lineage-specific functions. For instance, 30% of human lncRNAs are estimated to be primate-specific [45]. We note that the current evolutionary profile of lncRNAs does not indicate lack of function. Lack in overall conservation could be explained by fewer interactions of these lncRNAs with other molecules (e.g., proteins), where the remainder of the transcript would be free to explore new regulatory roles, resulting in rapid evolution [46]. Thus, while phylogenetic analysis can be used to help verify secondary structures of lncRNAs derived from experimental data, it is difficult to determine lncRNA structures from this analysis alone.

Individual 2-D methods or combinations of these methods have been applied to produce the secondary structures of many housekeeping RNA molecules [42,43]. These methods have also been used to study viruses, UTRs and introns of mRNA molecules [20,47–50]. Recently, using a variety of 2-D approaches, we produced the first experimentally-derived secondary structure of a human lncRNA, the steroid receptor RNA activator (SRA) [51]. SRA modulates the hormone receptor-signaling pathway [12], directly interacting with the estrogen receptor and many other co-activator and co-repressor factors [52,53]. It has been suggested that SRA acts as scaffolding in the final nuclear receptor assembly. Our experiments reveal a complex 2-D organization of this transcript, consisting of four major subdomains. It comprises >20 helical regions with a variety of terminal and internal loops. We find that the single-stranded regions are purine-rich, a common feature observed in other RNAs [54]. In addition, we employed the Shot-Gun Secondary Structure determination method by probing isolated fragments of SRA transcript in addition to the full transcript [51]. This approach allowed us to validate the modularity of a number of the SRA sub-regions.

## 4. 2-D RNA Structure Predictions

Thousands of new lncRNA molecules have been discovered in recent years. Traditional experimental investigations of RNA structure—one molecule at a time—cannot cope with this large demand. Many powerful computational methods have been developed to produce predictions of RNA secondary structures (Figure 3). These include Mfold, RNAstructure, the Vienna RNA package and others [55–58]. The majority of these tools are based on the thermodynamics parameters for base-pairing, base-stacking and simple hairpins. Many other parameters, such as kinetics of RNA folding, non-canonical interactions between nucleotides, long-range tertiary contacts and specific RNA motifs are challenging to account for and adapt to real experimental conditions. These tools are proven to be accurate for short RNA sequences (<100 nts), but the accuracy drops with the increase of RNA length [59]. The class of lncRNA is above 200 nts, where some achieve >100 kB. A key challenge lies in extending these methods to produce highly accurate predictions for very long sequences such as lncRNAs.

Many RNA prediction programs incorporate sequence homology to improve their structure prediction accuracies. These programs include RNAalifold, Construct, CM-finder and others [57,60–62]. It is challenging to apply these methods to lncRNAs, due to the lack of diverse sequences and lack of conservation of sequence. A current trend is to integrate experimental probing data with computational strategies. For example, the RNAstructure package incorporates SHAPE probing results as pseudo-free energy constraints in the energy minimization algorithm [63]. New high-throughput structure studies of the transcriptome (discussed below) are in need of tools capable of analyzing thousands of transcripts simultaneously, while at the same time being robust to sparse sampling. The integrative SeqFold package has been developed to tackle the RNA structurome. This package is based on Boltzman-weighted sampling to decrease the sensitivity to noise [64]. It has been adapted to handle the diverse sets of experimental data from enzymatic to chemical probing. SeqFold showed improved accuracy over other methods for a set of short RNA transcripts. Our recent structure of SRA presents an interesting benchmark that could be used to test this and other methods on longer RNA molecules [51]. We note that many excellent comprehensive descriptions and performance surveys of RNA structure prediction methods have been published previously [58,65–67].

## 5. Approaches to Determine 3-D Structures

Methods used to gain information about RNA tertiary contacts include UV/chemical crosslinking and hydroxyl radical probing [68–70]. X-ray crystallography and NMR provide 3-D atomic resolution structures of RNA molecules (Figure 3) [71–73]. X-ray crystallography is an intensive process, where robust homogenous RNA systems are successful candidates for crystallization. NMR can also be employed to produce atomic-level information about RNA structure and is excellent for studying particular RNA motifs [73]. For tens of thousands of transcripts, applying these tools in a high-throughput fashion is a significant challenge. In the case of lncRNAs, there are many basic structural questions that remain unanswered. Do lncRNAs have the potential to adopt higher-order tertiary organization, especially in light of their rapid evolution? Do lncRNAs sample many tertiary configurations? How stable are these tertiary interactions? In addition to providing mechanistic information, secondary structure studies lay the foundation for more labor-intensive 3-D studies by demonstrating that particular lncRNAs are structured.

## 6. Genome-Wide RNA Structural Studies and Sequencing Platforms

Until recently, only low-throughput structural studies of RNA molecules—one molecule at a time—have been published and accessed. Structural investigations of entire transcriptomes have been realized with high-throughput sequencing platforms [74]. Two independent pilot papers, which study the structures of thousands of RNAs, were published in 2010. One introduced the method of Parallel Analysis of RNA Structure (PARS). This method utilizes enzymatic probing to study all yeast mRNAs simultaneously [75]. Structural data for more than 3000 transcripts were obtained in a single experiment. PARS uncovered a number of general features for yeast mRNAs. For example, the coding regions of mRNAs exhibit stronger pairing than UTR regions. In addition, the codons of mRNAs show the three-nucleotide periodicity in their structure, where the first and the second nucleotides of each codon are the least and the most structured, respectively. While it is not clear whether these mRNA features are yeast-specific or are conserved across species, the studies raise new questions and will fuel new avenues of research. The PARS methodology was further extended to probe mRNA structure at higher temperatures to investigate their melting profiles and response to heat-shock [76].

The second paper introduced FragSeq approach, utilizing RNase P1 (a single-stranded RNA nuclease) to analyze the structural content of nuclear ncRNA in mouse cell lines [77]. In both the PARS and FragSeq approaches, the protocol of library construction is based on the generation of 5′-monophosphate transcripts, resulting from RNase cleavage. The PARS methodology includes an additional step of alkaline hydrolysis to shorten the transcripts, while FraqSeq does not. Therefore, the FraqSeq pool comprises short ncRNAs, resulting in increased sampling and coverage. 5′-monophosphate RNA fragments are further size-selected, ligated to adaptors, reverse transcribed, amplified by PCR and sequenced on the ABI SOLiD platform. Both PARS and FragSeq have been performed on *in vitro* refolded RNAs.

While the above protocols rely on RNase digestion to generate smaller RNA fragments, other RNA chemical probing protocols, which modify RNA, can be adapted to deep-sequencing platforms (SHAPE-seq) [78]. The development of these methodologies moves us one step closer to massive structural interrogation of entire transcriptomes *in vivo*.

## 7. The Next Step: *In Vivo* RNA Structure Determination

Ideally, we need to study RNA structure in its natural environment: living cells. Because RNA folds while being transcribed, the rate of transcription is an important parameter for consideration [79]. In cells, multiple factors play significant roles in the determination of the RNA fold. These include crowding, RNA-protein interactions, and local cellular conditions such as temperature, pH, stress and deprivation of nutrients [80–83]. Despite a large number of available chemical reagents developed for *in vitro* applications, only the base-specific DMS reagent was applicable to study intact cells and has been used for decades [84,85]. Chang and co-workers have adapted the SHAPE probing methodology to *in vivo* environments by developing two new SHAPE reagents [86]. These two reagents include FAI (2-methyl-3-furoic acid imidazolide) and NAI (2-methylnicotinic acid imidazolide). FAI and NAI are electrophiles with extended half-lives and better solubility. Their reactivities have been evaluated on the 5S rRNA in different cell lines, displaying reasonable modification profiles and consistent results. Moreover, these reagents were successfully tested in modifying nuclear RNAs, in particular, a small nucleolar RNA (SNORD3A) and the U2 RNA. These results suggest that lncRNAs, primarily nuclear-retained transcripts, can be successfully investigated by using these reagents in the future.

## 8. Conclusions

Chemical probing is the only available technology for RNA structure determination that has no restrictions in terms of the size of RNA molecules, their quantity and heterogeneity. Most importantly, chemical probing is not constrained to *in vitro* analysis. This is the only method that can provide an experimental structural output for any given RNA sequence *in vitro* and *in vivo* at the nucleotide level of detail [86]. Moreover, this method can be adapted to high-throughput sequencing platforms. Therefore, substantial efforts have been applied to develop new *in vivo* chemical probing reagents, new library construction protocols for sequencing platforms and improved RNA prediction software based on experimental data. Significant advances have been made in the past two years.

The advancement of *in vivo* RNA analysis tools is likely to produce an explosion of RNA structural information in the near future. The next step will be to determine the particular structural elements that are functional. One approach is to analyze lncRNA sequence conservation in the context of experimentally-determined secondary structures. This route can uncover common motifs and regulatory trends conserved at the structural level, which were previously overlooked. In light of the large amount of data, this task will be an interesting challenge for bioinformatics investigators.

Without doubt, coupling chemical *in vivo* reagents with deep-sequencing platforms offers new research directions in studying entire structurome not only under normal conditions, but under stress, changes in temperature and other variations of environment [64,74,75,77,86]. Thus, another route is to perform the investigations of the RNA structurome in various experimental conditions and examine differences that exist between various organisms. The observed differences in RNA structure can guide the researcher to new regulatory mechanisms of RNA in a genome-wide context. Such studies may also shed light on the disease-associated regulatory network and link the critical structural elements to development.
